# Supplementary material for: A Highly Expressed Antennae Odorant-Binding Protein Involved in Recognition of Herbivore-Induced Plant Volatiles in Dastarcus helophoroides
Source: Int J Mol Sci. 2023 Feb 9;24(4):3464. doi: 10.3390/ijms24043464 (PMC9962305; doi:10.3390/ijms24043464)
Supplement: Supplementary file 1 [file ijms-24-03464-s001.zip › Supplemental Figure S1.pdf]

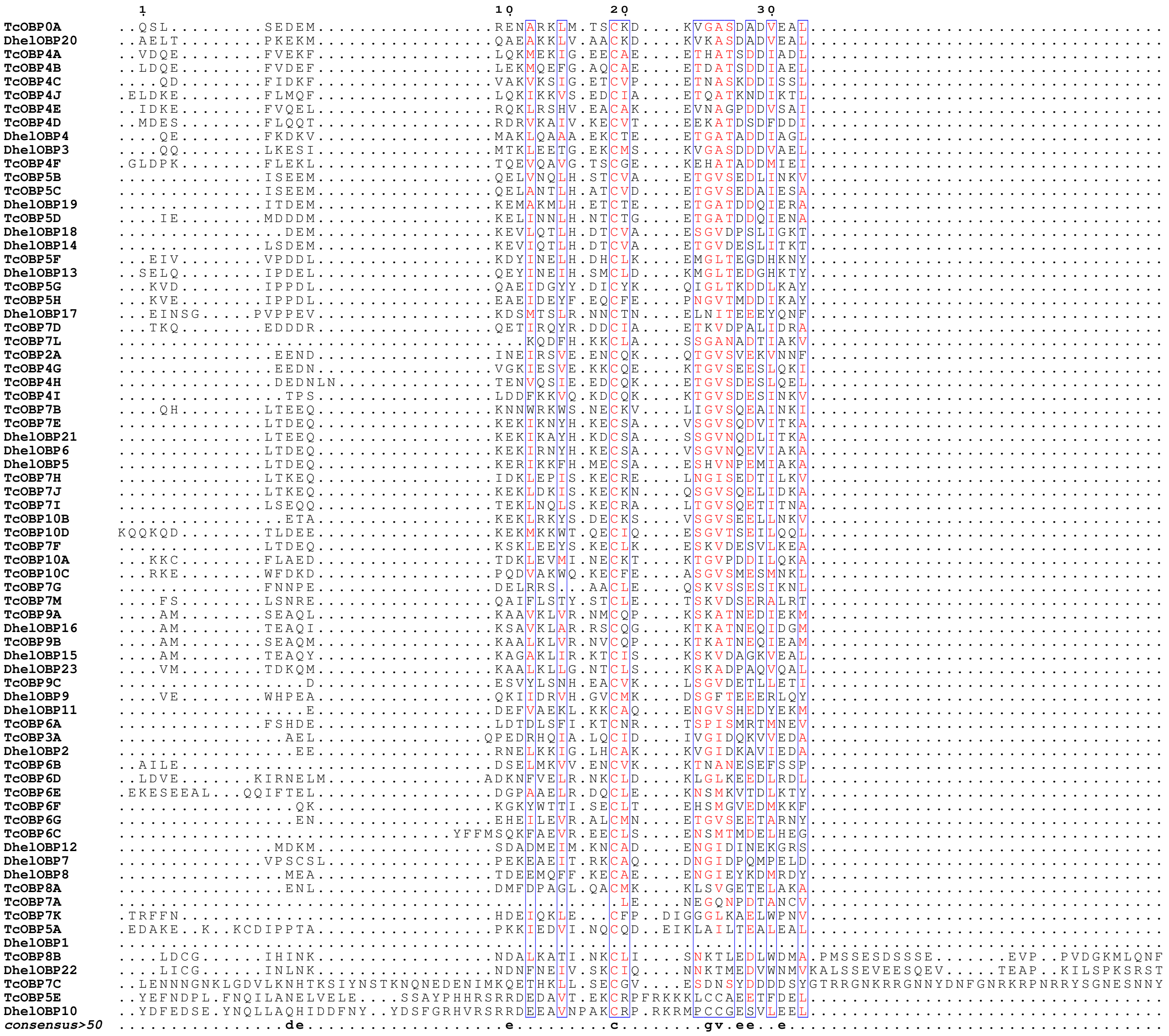

```
                                                             40
TcOBP0A          ...................KMHQMP.................E....SREGF................................................
DhelOBP20        ...................KAQKIP.................E....THEGL................................................
TcOBP4A          ...................IEQR.D................PK....THEGK................................................
TcOBP4B          ...................IARKLP................PS....THEGK................................................
TcOBP4C          ...................LAHK.M................PD....SHEGK................................................
TcOBP4J          ...................LEHK.I................PD....SHEGK................................................
TcOBP4E          ...................FAHK.L................PA....THEGK................................................
TcOBP4D          ...................MALK.I................PT....SHEGK................................................
DhelOBP4         ...................IAKK.P................PT....THEGQ................................................
DhelOBP3         ...................IARK.R................PS....RHEGM................................................
TcOBP4F          ...................MEEKFP................PT....SHEAK................................................
TcOBP5B          ...................NSDKVM................ID....DEKLK................................................
TcOBP5C          ...................RKGNFA................PD....DK.LK................................................
DhelOBP19        ...................RKGDFP................PN....DE.FK................................................
TcOBP5D          ...................RKGNFA.................E....DDSFK................................................
DhelOBP18        ...................MAEKKL................PE....DDKLK................................................
DhelOBP14        ...................MADKKL................PE....DDKLK................................................
TcOBP5F          ...................NIHVK..................(.)..DPKMM................................................
DhelOBP13        ...................NIDNK.......................DPKWM................................................
TcOBP5G          ...................KIGDR.......................DPKIM................................................
TcOBP5H          ...................KMGDK.......................DPKIM................................................
DhelOBP17        ...................DW.NW.......................EPKLM................................................
TcOBP7D          ...................DNGDFT.................D....DAKLQ................................................
TcOBP7L          ...................RNGKFS.................N....DPQTQ................................................
TcOBP2A          ......................ELV.................D....DPLVK................................................
TcOBP4G          ...................MRLEEV.................D....DPLVK................................................
TcOBP4H          ..................SETGDS..................D....DPLVK................................................
TcOBP4I          ...................NNLEPV................CD....DLLLQ................................................
TcOBP7B          ...................RNNEFD.................S...VDDKIK................................................
TcOBP7E          ...................RKGEFI.................E....DPKFK................................................
DhelOBP21        ...................RKGEFV.................E....DPKLM................................................
DhelOBP6         ...................RKGEFV.................E....DPKFK................................................
DhelOBP5         ...................RKGEFT.................E....DPSFM................................................
TcOBP7H          ...................RRGEAV.................N....EPKLK................................................
TcOBP7J          ...................RTGELI.................N....DPKLK................................................
TcOBP7I          ...................RNGNFE.................E....DPKLK................................................
TcOBP10B         ...................RNHEDV.................H....DPKLD................................................
TcOBP10D         ...................RNQKRV.................E....DPKLK................................................
TcOBP7F          ...................EKGVYL.................D....DPKLM................................................
TcOBP10A         ...................RNGEKI.................D....DPKLR................................................
TcOBP10C         ...................PNITLS.................E....DPKLG................................................
TcOBP7G          ...................QIGNFD.................D....DERLK................................................
TcOBP7M          ...................ASGIID.................D....EPKLK................................................
TcOBP9A          ...................HHGDW.................NI....DRTAM................................................
DhelOBP16        ...................HKGNW.................DI....DHTTM................................................
TcOBP9B          ...................HTGNW.................DL....DKNGK................................................
DhelOBP15        ...................RKGEW.................PE....EKALM................................................
DhelOBP23        ...................RKGEW.................PE....EKPIM................................................
TcOBP9C          ...................YEGDVF.................E....DMKFK................................................
DhelOBP9         ...................TFDN...................E....DRRFK................................................
DhelOBP11        ...................RTFVVV.................E....NDKAI................................................
TcOBP6A          ...................LINKKLG..............HGE....SSAFK................................................
TcOBP3A          ...................INIEIP................KN....NPKYK................................................
DhelOBP2         ...................VATAIL................PK...ENMKYR................................................
TcOBP6B          ...................NFLETT.....................PSQPAL................................................
TcOBP6D          ...................KFDGDV......................SEDLM................................................
TcOBP6E          ...................NTSNDI......................PEKEL................................................
TcOBP6F          ...................DLPAEK................MS.....EEML................................................
TcOBP6G          ...................KPAEDP.....................ASEEIL................................................
TcOBP6C          ...................WKMENL......................PESHL................................................
DhelOBP12        ........................................RSE..TPSPERQ................................................
DhelOBP7         .............................................MTSKDVQ................................................
DhelOBP8         ...................SFKDV.......................PEELH................................................
TcOBP8A          ...................LEDKSK................DP....PEKIM................................................
TcOBP7A          ..................ALGGQRI.....................KDSEIA................................................
TcOBP7K          ....................................................................................................
TcOBP5A          ...................NINEHTKS.......RAKRDTFSD....DEKRI...AG...........................................
DhelOBP1         ....................................................................................................
TcOBP8B          ...................RIKRASVR..LTNTE......TNE...TTPEPK.........AVSSEAQATEN............................
DhelOBP22        .................SRRSRRAASS..RRNTR...ERSTTE.ATTTPEPE...PEDDEQTTVETSTQVVEDG..........................
TcOBP7C          NRYGNRNRGNRYSGNDDENNSNESTNRGNRRNNGN.RNRYNNE.NYSDEEDS...NESNQSNNQRRYNRTMSNTGYYYGDGGYHNECNDNHGYRNQWRPF
TcOBP5E          ..................HDKDRDF...........KRECFKQVVGSKDGPR.EF.DPFRCDKVDKHRRDMT............................
DhelOBP10        ..................HDKDKDI...........RRQCFREIIGKDKEPRGEH.DPFRCDRIEKHRKEMT............................
consensus>50     ......................e...................e....d....................................................
```

```
                                                       50         60            70        80        90
TcOBP0A       ...................................CMLECVFDSAKIM.QDGKFSKS....GMIEGFKPLIGDDKAKLESLEKLSATCESELG.......
DhelOBP20     ...................................CLIECLFDGAKIM.QNGKFDKN....GMMTAFSAALKEQGKSPDKLKSLGDACEKDVGE......
TcOBP4A       ...................................CLIFCYHKKFNTMKEDGSLDKV....GSVLALEEVRDADFELYKNILTIFVTCGDKAK.......
TcOBP4B       ...................................CMIFCMQKKFNMMKENGGIDRA....GAIAALKPLQKADPELHQKVLKIFVTCGMRVK.......
TcOBP4C       ...................................CLIFCFHKQFQIQNDDGSINRE....GAIKALEPLKADDAELYEKVISIFKKC.ESTP.......
TcOBP4J       ...................................CMIFCFHKHFQIQNEDGSLNKV....AAISLLEPIKDHSQDIYDKVVKIFNTCFDSAE.......
TcOBP4E       ...................................CIFFCMHKLYNAQNEDGSLNMA....GALANLELIKDMDPDVYTKVSTSFKNC.ESAP.......
TcOBP4D       ...................................CVFFCSHKKFNMQHPDGSINKE....GALDTFEVVKDVDAEFHDKVITVYNHC.LSTP.......
DhelOBP4      ...................................CMIFCMHKIFKVQKDDGSTGGE....EAIKFLDPLKENDPALHDKMVQIYQTCANA.P.......
DhelOBP3      ...................................CMVHCFYEEFKIMDAEGNLSRE....ESLKLLEPVKADDPDLYGKVIVIAKKCKEAVP.......
TcOBP4F       ...................................CVVACFYKHYKMMKEDGTFDKD....AAVKAFDEIKAQDAEIHAKILKVIDACDAKKQ.......
TcOBP5B       ...................................CYIKCLLTETGCISDDGVVDVE.....ATIAL...LPEDMKA..KTTPVIRSCGAKM........
TcOBP5C       ...................................CYMKCIMEQMACIDDEGIIDVE.....ATIAV...LPEEYQA..KAEPIVRKCGTKI........
DhelOBP19     ...................................CYIKCILAQMACISDDGMIDVE.....ATIAV...LPEEFKA..DAEPIIRKCGTVV........
TcOBP5D       ...................................CYFKCVFDQMGCMTDDGKVDSE.....AVIAV...MPPELAD..KIASTVRGCTE..........
DhelOBP18     ...................................CYIKCLQAETGTL.EGGVMDVE.....GVLSF...VPEDVKP..KVEATIRKCGSKP........
DhelOBP14     ...................................CYIKCLQTETGTM.EDGVLDAE.....GVLAF...TPDDIRP..KVEATIRKCGSQP........
TcOBP5F       ...................................CYMKCLMTTSKWMNMDESIQYD.....FILSS...VHPAVKN..ILLPALDKCRDIPK.......
DhelOBP13     ...................................CYMQCLMMTSKWMAPDQEIQYD.....YIISS...AHPQVAD..LLVPAVNKCRNIQE.......
TcOBP5G       ...................................CFMKCVFVEAKWMDENENLQYD.....YIKNT...IHHSIRH..ITLPELENCGKKAE.......
TcOBP5H       ...................................CFMRCLFVSGKWMDENENMQYD.....YIKET...IHHAIRH..ITIPELENCGKEAQ.......
DhelOBP17     ...................................CYIQCVLLNLGWITEENVPDFN.....AMEST...LPITYKS..YVDLNREHCHNVPD.......
TcOBP7D       ...................................CFSKCFYQKAGFVSETGDLLFD.....VIKDK...IPKEANRE.KALAIIDKCKELK........
TcOBP7L       ...................................KYFGCMLRSVGVVNQAGQLQVA.....ALRKQ...VPKDMKRD.EAMKIYMSCKDKK........
TcOBP2A       ...................................ENALCILKAYGIMDEDGNIYED.....KLKEQ...ITSELGEK.NAEQVAKKCTIKK........
TcOBP4G       ...................................ENALCTLKAYGVMDDDGNIFPD.....KFEEK...LKPEIGAD.EAKRVAEKCAVKK........
TcOBP4H       ...................................KNALCILKAYGVIDDQGEISED.....KLEEK...LEPDRGKE.EAEKVAKSCAVKK........
TcOBP4I       ...................................ENALCILKTYEVMDEEGKICPD.....KLMEV...LEPKFGKE.KAEKLIEKCTLEK........
TcOBP7B       ...................................KHGLCFAKKASLADSSGNIIIN.....QIKIK...LKRVIEDDEEVDRIVTKCTIRK........
TcOBP7E       ...................................EHLFCFSKKAGFQNEAGDFQEE.....VIRKK...LNAELNDLDATNKLIAKCAVKK........
DhelOBP21     ...................................EHLFCFSKKAGFQNEAGDIQTD.....VIKAK...LGAEIKDSATVDQLIKKCAIKK........
DhelOBP6      ...................................EHLFCFSKKVGFQNEAGDLQVE.....VLKAK...VGAEHKDQAVVDQLIKKCAVKK........
DhelOBP5      ...................................KHLFCFAKKAGLQNDAGEIQVD.....VLRAK...LGAELKDDAATDKLISMCAIQK........
TcOBP7H       ...................................NHVLCVSKKTGLASETGETNVE.....VLRTK...LRKVSENDDEVNSIIQKCVVKK........
TcOBP7J       ...................................AQIYCVSKKAGLATEAGEINMD.....NLKTK...LKKVAANDDEVNKIIQKCVVKK........
TcOBP7I       ...................................LQVLCIGKKVGIMNESSQIDEN.....VLKAK...LRKVSDNDEEVNKIYNKCAVKK........
TcOBP10B      ...................................EHGFCILKKAGFMNEAGDILAD.....TIKTK...LKENSEHPDTVDALVEKCNEKK........
TcOBP10D      ...................................EYTFCTFKKNGFMNEDGKLQYD.....VIKST...LMKVSGSEEEANKVVKDCVVEK........
TcOBP7F       ...................................NHVYCLVKKINSQKDKGELEVT.....QIKEK...LMMQINDEKEVDKLIQLCLVQE........
TcOBP10A      ...................................EHALCMMKKSEMMNDAGEMQMD.....KIRAR...IKHAVSNEAEGTRIMNECAVKK........
TcOBP10C      ...................................ENAFCLLKKLGFISEDGTLLIE.....KLRTS...LKNQWGDE.IANKLVNECARQK........
TcOBP7G       ...................................EYLFCVSKNAGYQDPAGHLQHE.....MIRLR...FKGGRYSDDTINEVLQQCGHQK........
TcOBP7M       ...................................EFLFCINKQNGVQDDAGNFVKD.....AVRKR...IEHPLLTDKTMEIIVNKCTRKR........
TcOBP9A       ...................................CYMHCALNSNKLITKENVFNRDY.AITLAEKN...LPTALKT..ASIEAANLCKDSAK.......
DhelOBP16     ...................................CYLNCALAMYKLQRADNTFNLE..ASAAQLKQ...LPESLRE..ASAKTTESCKDSAK.......
TcOBP9B       ...................................CYMWCILNMYKLIGKDNSFDWEA.GIATLKAQ...APESVRD..PAIASVNNCKDAVK.......
DhelOBP15     ...................................CYLYCVLASYKVVTPENTLDVEN.GVKALNAQ...APESIRD..AAIISTKNCKDSAK.......
DhelOBP23     ...................................SYLYCVLNTQNIITKES..............................GACAN..........
TcOBP9C       ...................................TYIHCFFKKSGFQDENGVMHFD.....AIKSS...FHKDFSQTENIDKTITECEEKKL.......
DhelOBP9      ...................................CYMYCILKEIDLVDNKDNVLPL....ESFMER...VVEHHKN..DFLPHAEICIPKVT.......
DhelOBP11     ...................................KTMVCLYKNIGFMDPKGKLQQS....GVIKIH...VEKLSMSKKEADQLATKCIKEGQ.......
TcOBP6A       ...................................CFLHCLFMKYGWMDSDGGFLLH.....DIKQT...LEESDVEIASLEFILYKCTATE........
TcOBP3A       ...................................EFLACSYKKQGYQNENGEILME.....NIKKF...LQKFYHP..SDLQELNSCSGHN........
DhelOBP2      ...................................EFLACSYKKQGYQDEEGHILYE.....NIKKF...LSKFYKE..SDLRAIDTCKETP........
TcOBP6B       ...................................CTAKCLLESLEIVNSEGNINME.....TLKEY......AQPFESPAREAVATCGEEIK.......
TcOBP6D       ...................................CFGKCIQEEDGLLDSEGNLNEE.....KLEKKIETMPFLSRVSDDTKNNIMECLKEIG.......
TcOBP6E       ...................................CFYKCFYEGVEFIDANGNLNVN.....NMKE....IPAISELGDEVLNEITACVEKIG.......
TcOBP6F       ...................................CFNKCFYDKLLITDENGEINTD.....NLMS....IPLVNAIDASKHDDLVTCLKKVG.......
TcOBP6G       ...................................CMVKCIFEKIGCLKDDGSFCVD.....TMKKK...NYIMDVINEENEEKIYECLRGVG.......
TcOBP6C       ...................................CFLKCLLEKREVIDENGVPQKE.....KIDE....ILTVKQLSDEKREEISTCITNVE.......
DhelOBP12     ...................................CFLKCLLEKREIIDKDGKVDEQ.....KVKLK......HQAMGMELDDETFECLKKLP.......
DhelOBP7      ...................................CALKCILEQGNYVDESGKILVE.....AFKQY......DIKIGKVNEEESYECLQKLP.......
DhelOBP8      ...................................CYIKCFAEKGGYLDDNGKFNIE.....KARAR......DHGNGEEALEKAYQCLENLS.......
TcOBP8A       ...................................CLFKCALEDSGFL.QDGVVDKS.....KW.....................PMPECVQDVV.......
TcOBP7A       ...................................KMAHCILTKTNLMTDKGTFNSN.....LLKER...LRQSVHSDELVDKVVMMCTVEKE.......
TcOBP7K       .......................................VSCVFHRKGFTDDKGEFKID.....VLKQK...LSKFQDDKYLVNEIAELCVDKHD.......
TcOBP5A       ...................................CLLQCVYRKMKAVNEKGFPTVE..GLVALYSE...GVTQKEYIIATLQAVNVCLNKAQKKHLTKP
DhelOBP1      ............................................MNAVNQHGFPTVE..GLVALYSE...GVTQKEYILATLQSVTYCLSAAQKKYLITP
TcOBP8B       ...................................CIIQCIFDNLQMTDSTGYPVHTKILDGLLKNT...TNRELRD..FLQDTTDECFQVMDK......
DhelOBP22     .................................KGCFVYCIFQNLGMTGDNGFPQLPKITDGLLKSA...TGKELKD..FLQDSADECYQEAIK......
TcOBP7C       GGNVGYDYGNMRRGYGRNLDMNLRAKRSNDNDDSQCVSQCVFGYLEVLDDNRVPSETL....VIKWLQDHLSNDMKRI.RALREARRCFARLST......
TcOBP5E       ...................................CVSQCVGQKKDVLDKDGNVKEAE.FGEFVKET...MAKESWFVSIQDKVVSTCLAEARNA...TA
DhelOBP10     ...................................CVMQCVEQKKDLLDESGNVNEEE.FAKFVSET...FEKEAWRA......................
consensus>50  ...................................c...c........dedg..d.e................d.............c............
```

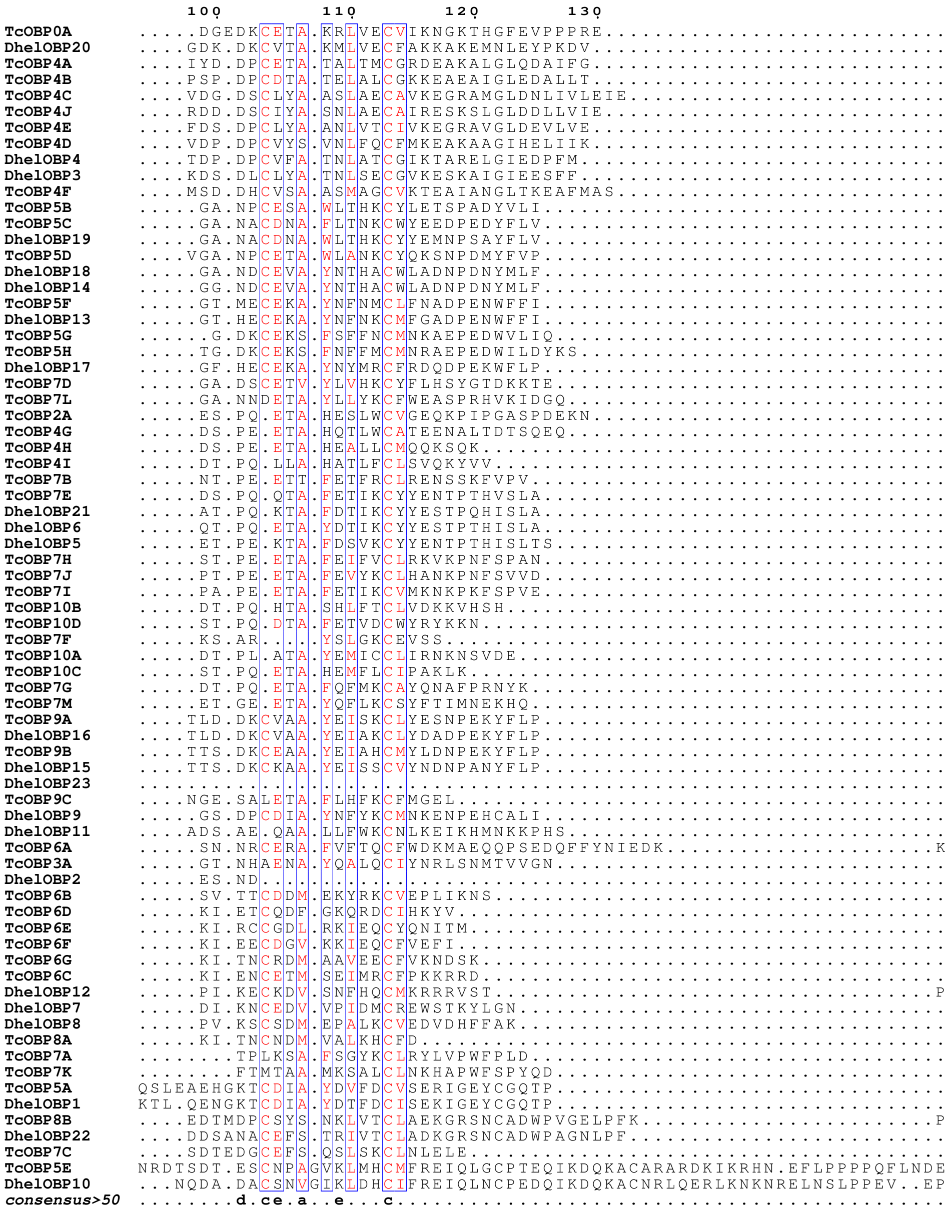

Figure S1 Mutiple sequence alignment of OBPs from *Dastarcus helophoroides* and *Tribolium castaneum*
