# Supplementary material for: A Highly Expressed Antennae Odorant-Binding Protein Involved in Recognition of Herbivore-Induced Plant Volatiles in Dastarcus helophoroides
Source: Int J Mol Sci. 2023 Feb 9;24(4):3464. doi: 10.3390/ijms24043464 (PMC9962305; doi:10.3390/ijms24043464)

DhelOBPs' mRNA expression levels in antennae of male adults

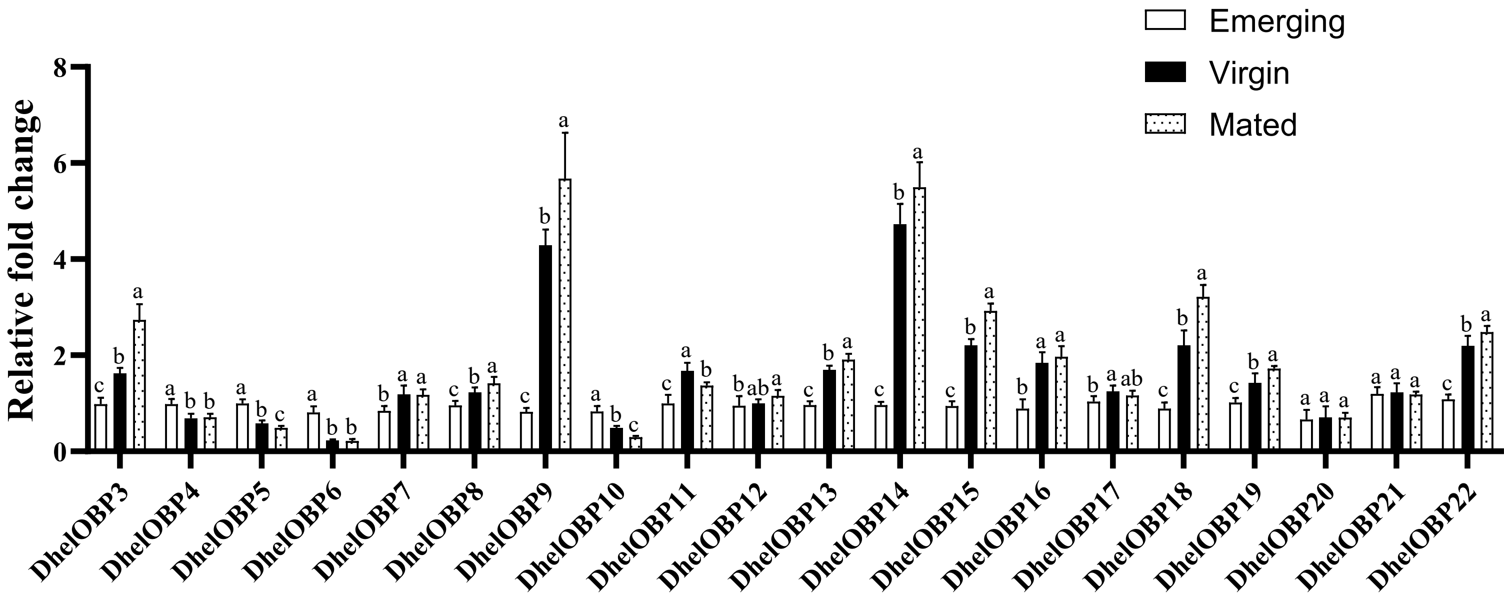

DhelOBPs' mRNA expression levels in antennae of female adults

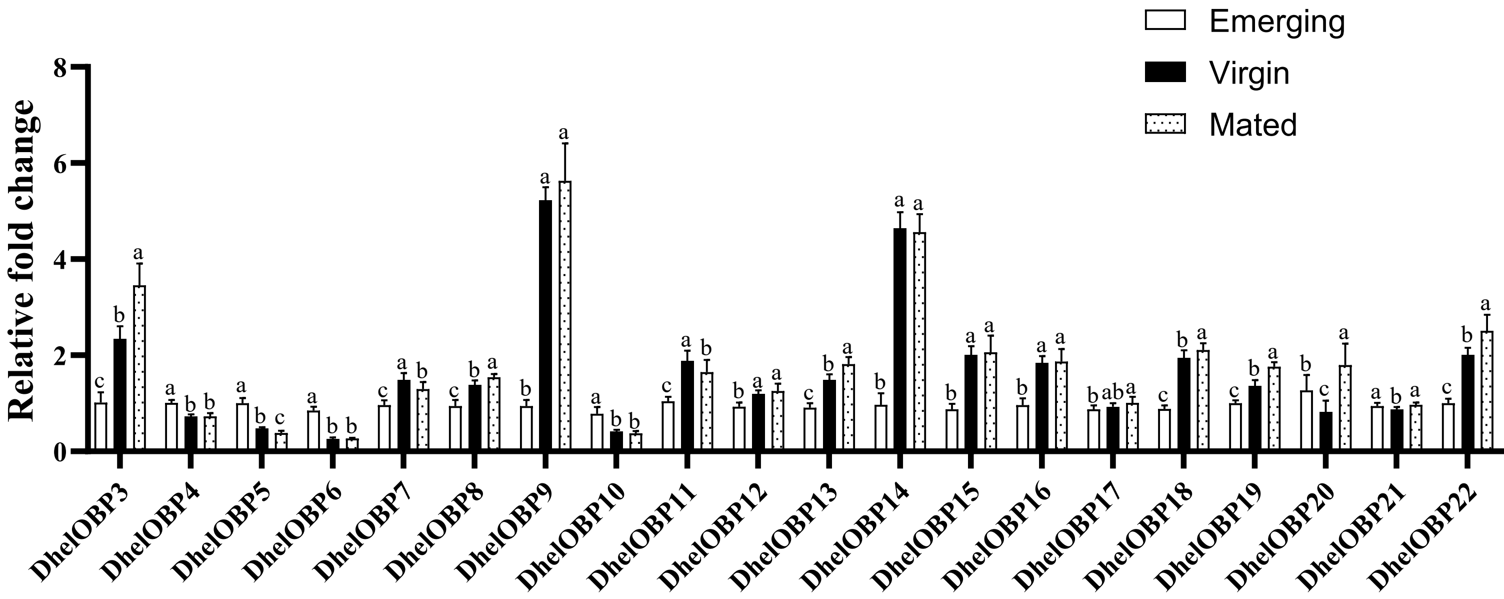

Supplement: Supplementary file 1 [file ijms-24-03464-s001.zip › Supplemental Figure S2.pdf]
