# Supplementary material for: A Highly Expressed Antennae Odorant-Binding Protein Involved in Recognition of Herbivore-Induced Plant Volatiles in Dastarcus helophoroides
Source: Int J Mol Sci. 2023 Feb 9;24(4):3464. doi: 10.3390/ijms24043464 (PMC9962305; doi:10.3390/ijms24043464)
Supplement: Supplementary file 1 [file ijms-24-03464-s001.zip › Supplemental Figure S4.pdf]

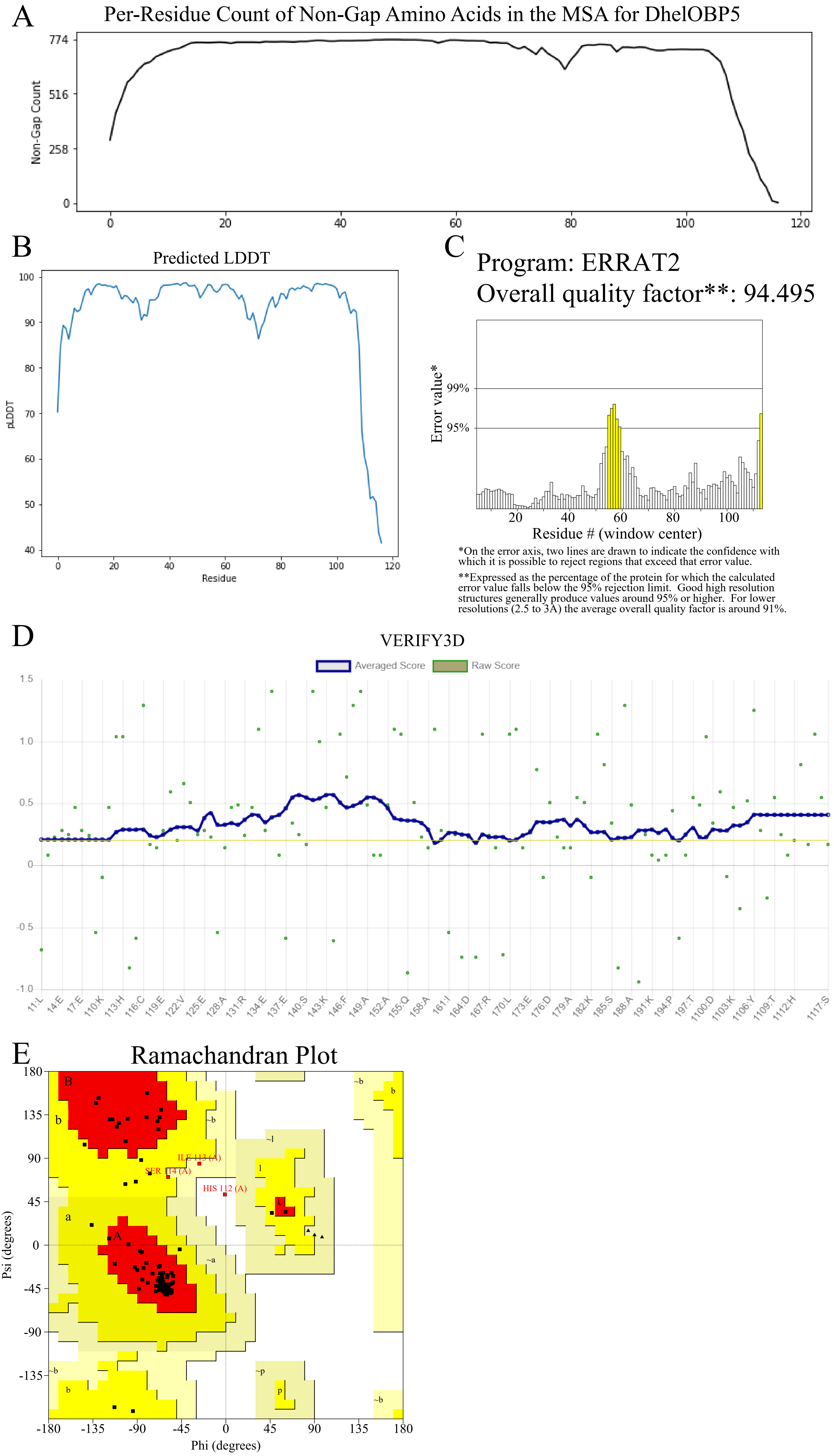

Plot statistics

Residues in most favoured regions [A,B,L]	94	87.9%
Residues in additional allowed regions [a,b,l,p]	10	9.3%
Residues in generously allowed regions [~a,~b,~l,~p]	2	1.9%
Residues in disallowed regions	1	0.9%
	----	------
Number of non-glycine and non-proline residues	107	100.0%
Number of end-residues (excl. Gly and Pro)	2	
Number of glycine residues (shown as triangles)	4	
Number of proline residues	4	

Total number of residues	117	

Figure S4. Evaluation of DhelOBP5 3D modeling. (A) Mutiple sequence alignment of DhelOBP5. (B) Predicted local distance difference test (pLDDT) on a scale from 0 to 100. (C-E) The qualities of 3D model were evaluated by ERRAT, VERIFY 3D and PROCHECK programs (https://saves.mbi.ucla.edu/).
