# Supplementary material for: A Highly Expressed Antennae Odorant-Binding Protein Involved in Recognition of Herbivore-Induced Plant Volatiles in Dastarcus helophoroides
Source: Int J Mol Sci. 2023 Feb 9;24(4):3464. doi: 10.3390/ijms24043464 (PMC9962305; doi:10.3390/ijms24043464)
Supplement: Supplementary file 1 [file ijms-24-03464-s001.zip › Supplemental Figure S6.pdf]

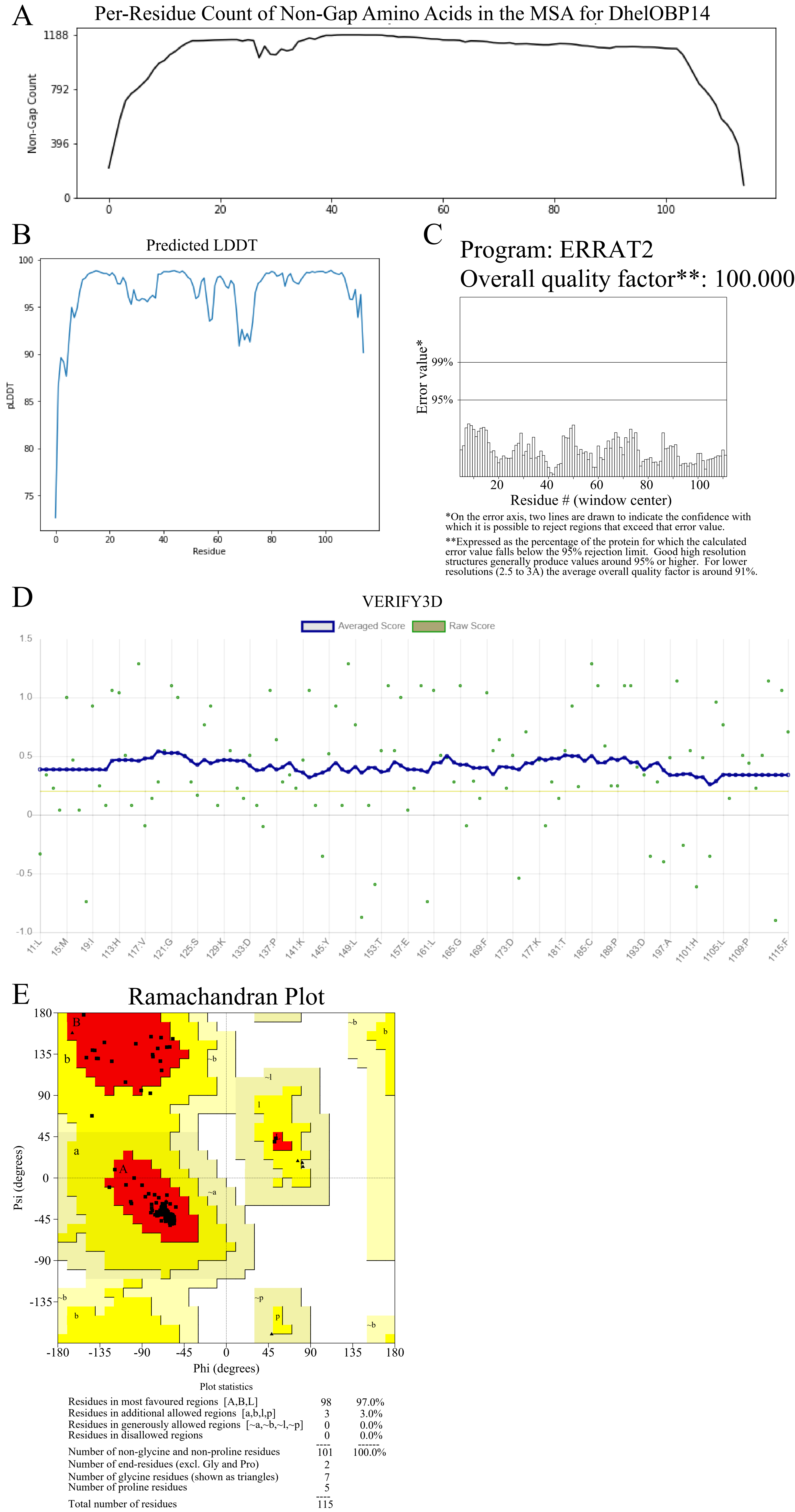

Figure S6. Evaluation of DhelOBP14 3D modeling. (A) Mutiple sequence alignment of DhelOBP14. (B) Predicted local distance difference test (pLDDT) on a scale from 0 to 100. (C-E) The qualities of 3D model were evaluated by ERRAT, VERIFY 3D and PROCHECK programs (https://saves.mbi.ucla.edu/).
