# Supplementary material for: A Highly Expressed Antennae Odorant-Binding Protein Involved in Recognition of Herbivore-Induced Plant Volatiles in Dastarcus helophoroides
Source: Int J Mol Sci. 2023 Feb 9;24(4):3464. doi: 10.3390/ijms24043464 (PMC9962305; doi:10.3390/ijms24043464)
Supplement: Supplementary file 1 [file ijms-24-03464-s001.zip › Supplemental Table S1.pdf]

Table S1. Information of volatiles from *Pinus massoniana* Lamb wood sections

Compound	Retention Time (min)	PubChem CID	CAS Number
α-Pinene	8.912	6654	80-56-8
α-Fenchene	9.260	28930	471-84-1
Camphene	9.308	6616	79-92-5
β-Pinene	10.117	14896	127-91-3
Myrcene	10.417	31253	123-35-3
p-Cymene	11.454	7463	99-87-6
D-Limonene	11.612	440917	5989-27-5
β-phellandrene	11.652	11142	555-10-2
γ-Terpinene	12.389	7461	99-85-4
Terpinolene	13.178	11463	586-62-9
Fenchone	13.312	14525	7787-20-4
Fenchol	14.145	15406	1632-73-1
Camphor	14.939	2537	76-22-2
Pinocamphone	15.272	6427105	547-60-4
Isopinocamphone	15.707	84532	14575-93-0
Terpinen-4-ol	15.820	11230	562-74-3
α-Terpineol	16.186	17100	98-55-5
(-)-Verbenone	16.519	92874	1196-01-6
α-Longipinene	20.146	520957	5989-08-2
Ylangene	20.585	20055075	14912-44-8
Longicyclene	20.754	564934	1137-12-8
(+)-Sativene	21.138	11275742	3650-28-0
Longifolene	21.706	289151	475-20-7
trans-Caryophyllene	21.842	5354499	87-44-5
β-Farnesene	22.337	5281517	18794-84-8
Humulene	22.587	5281520	6753-98-6
