# Supplementary material for: A Highly Expressed Antennae Odorant-Binding Protein Involved in Recognition of Herbivore-Induced Plant Volatiles in Dastarcus helophoroides
Source: Int J Mol Sci. 2023 Feb 9;24(4):3464. doi: 10.3390/ijms24043464 (PMC9962305; doi:10.3390/ijms24043464)
Supplement: Supplementary file 1 [file ijms-24-03464-s001.zip › Supplemental Table S3.pdf]

Table S3. Primers and amplification efficiencies for qRT-PCR

Primers	Base sequence	Amplification efficiency
RPS-QF	AAAATGTGGGCTACACCAAAGA	0.8854
RPS-QR	GAAAAGCACTCGCAAAAATGGC	
GAPDH-QF	TAACTTTGGCATCGTTGAGG	0.9111
GAPDH-QR	AGCGGCGGGAATGATGTTTT	
Tubulin-QF	TCGGTGGTGGTACTGGGTCT	0.8255
Tubulin-QR	ACGGCTGTTGAAACTTGAGGA	
DhelOBP3-QF	TGATTTTTGCGACCCTTGTGG	0.8238
DhelOBP3-QR	CCTCATGTCTCGAAGGTCGT	
DhelOBP4-QF	GCCACCAACAACCCATGAAG	0.9304
DhelOBP4-QR	TTTTGTCATGCAGAGCGGGA	
DhelOBP5-QF	CATGGAGTGCTCGGCTGAAA	0.8744
DhelOBP5-QR	CCCGCGTCATTTTGTAGTCC	
DhelOBP6-QF	CAAGGCTCGTAAGGGGGAAT	0.8454
DhelOBP6-QR	TCCTTATGTTCAGCGCCCA	
DhelOBP7-QF	TCTTGGGCAGTTCCATCTTGT	0.9781
DhelOBP7-QR	CAGGGCGCACTGTACATCTTT	
DhelOBP8-QF	GCCGAGAAAGGGGGTTACTT	0.8278
DhelOBP8-QR	AGAGCTGGTTCCATATCGCTG	
DhelOBP9-QF	CTTGGGCAACATTCAAGGGG	0.8785
DhelOBP9-QR	GTGTATTGCAGGCGTTCCTC	
DhelOBP10-QF	AAAAGAATGCCGTGTTGCGG	0.8673
DhelOBP10-QR	TCATGTTCACCTCTCGGCTC	
DhelOBP11-QF	CGCAGAGGATGAATTTGTAGCA	0.8036
DhelOBP11-QR	AGCCAGTTGGTCAGCTTCTTT	
DhelOBP12-QF	AGATCCCGTTCCGAAACTCC	0.9823
DhelOBP12-QR	CATCATCCAGTTCCATCCCCA	
DhelOBP13-QF	ATCTAGTGCCCATCCCCAAG	0.8554
DhelOBP13-QR	TCACATTCGTGGGTTCCTTCT	
DhelOBP14-QF	TGTTGCGGAAACCGGAGTT	0.9085
DhelOBP14-QR	CGGTCTGATATCGTCTGGTGT	
DhelOBP15-QF	TGTATTGCGTTCTGGCATCCT	0.8887
DhelOBP15-QR	TTATGGCAGCGTCTCTGATGG	
DhelOBP16-QF	GCCAAGGAAAAACCAAGGCA	0.9290
DhelOBP16-QR	AGGTGTTATCCGCCCTTTGC	
DhelOBP17-QF	CACCACCTTCGCCAATTCAG	0.8039
DhelOBP17-QR	TGGGCTCCCAATTCCAATCAA	
DhelOBP18-QF	GACAGGAACGTTAGAGGGTGG	0.8198
DhelOBP18-QR	CCTCGCAATCATTTGCTCCAG	
DhelOBP19-QF	TGTTACGGGCATTACAGACGA	0.8559
DhelOBP19-QR	TCGCCTTTTCGGGCTCTTTC	
DhelOBP20-QF	AAATGGCATGATGACCGCCT	1.1245
DhelOBP20-QR	TCCTTGTCGCCTTCACCTAC	
DhelOBP21-QF	TCATCACCAAAGCACGCAAAG	0.8337
DhelOBP21-QR	TGGATATCACCGGCTTCGTT	
DhelOBP22-QF	GAAAGATCGACCACGGAAGC	0.8343
DhelOBP22-QR	TCGACGTTTCCACCGTAGTTT	
