# Supplementary material for: A Highly Expressed Antennae Odorant-Binding Protein Involved in Recognition of Herbivore-Induced Plant Volatiles in Dastarcus helophoroides
Source: Int J Mol Sci. 2023 Feb 9;24(4):3464. doi: 10.3390/ijms24043464 (PMC9962305; doi:10.3390/ijms24043464)
Supplement: Supplementary file 1 [file ijms-24-03464-s001.zip › Supplemental Table S4.pdf]

Table S4. IC50 and K_i of the tested HIPVs to DhelOBPs

Ligands	DhelOBP4				DhelOBP5				DhelOBP6				DhelOBP14				DhelOBP18				DhelOBP20			
	pH 7.4		pH 5.0		pH 7.4		pH 5.0		pH 7.4		pH 5.0		pH 7.4		pH 5.0		pH 7.4		pH 5.0		pH 7.4		pH 5.0	
	IC50	K_i	IC50	K_i	IC50	K_i	IC50	K_i	IC50	K_i	IC50	K_i	IC50	K_i	IC50	K_i	IC50	K_i	IC50	K_i	IC50	K_i	IC50	K_i
p-Cymene	13.0 ± 0.9	12.0 ± 0.9	> 50	-	> 50	-	ud	ud	ud	ud	ud	ud	> 50	-	> 50	-	> 50	-	> 50	-	> 50	-	> 50	-
γ-Terpinene	11.0 ± 0.3	10.1 ± 0.3	> 50	-	> 50	-	> 50	-	ud	ud	ud	ud	> 50	-	> 50	-	> 50	-	> 50	-	> 50	-	> 50	-
Terpinolene	15.7 ± 1.8	14.5 ± 1.7	> 50	-	> 50	-	> 50	-	ud	ud	ud	ud	> 50	-	> 50	-	> 50	-	> 50	-	> 50	-	> 50	-
Fenchone	23.8 ± 5.1	22.0 ± 4.7	47.4 ± 2.6	43.8 ± 2.4	> 50	-	> 50	-	ud	ud	ud	ud	> 50	-	> 50	-	> 50	-	> 50	-	> 50	-	> 50	-
Camphor	18.0 ± 2.4	16.6 ± 2.2	> 50	-	> 50	-	> 50	-	ud	ud	ud	ud	> 50	-	> 50	-	> 50	-	> 50	-	> 50	-	> 50	-
Terpinen-4-ol	27.8 ± 3.5	25.7 ± 3.2	> 50	-	> 50	-	> 50	-	ud	ud	ud	ud	> 50	-	> 50	-	> 50	-	> 50	-	> 50	-	> 50	-
α-Terpineol	10.4 ± 0.1	9.6 ± 0.1	> 50	-	> 50	-	ud	ud	ud	ud	ud	ud	> 50	-	> 50	-	> 50	-	> 50	-	> 50	-	> 50	-
(-)-Verbenone	23.1 ± 3.9	21.3 ± 3.6	> 50	-	> 50	-	ud	ud	ud	ud	ud	ud	> 50	-	> 50	-	> 50	-	> 50	-	> 50	-	> 50	-

Notes: IC50, ligand concentration displacing 50% of the fluorescence intensity of the DhelOBPs/1-NPN complex; K_i, dissociation constant (μM); '-' indicated the binding affinities were too weak (IC50 > 50 μM) to calculate accurate K_i values; 'ud' indicated an abnormal fluorescence intensity (increasing) that K_i values could not be calculated.
