# Supplementary material for: A Highly Expressed Antennae Odorant-Binding Protein Involved in Recognition of Herbivore-Induced Plant Volatiles in Dastarcus helophoroides
Source: Int J Mol Sci. 2023 Feb 9;24(4):3464. doi: 10.3390/ijms24043464 (PMC9962305; doi:10.3390/ijms24043464)
Supplement: Supplementary file 1 [file ijms-24-03464-s001.zip › Supplemental Table S5.pdf]

Table S5. Primers for homologous recombination of target *DhelOBPs*

Primers	Base sequence
DhelOBP4-HF	TATTTTCAGGGATCCGAATTC CAAGAGTTCAAAGACAAAGTAATGGC
DhelOBP4-HR	GTGGTGGTGGTGGTGCTCGAG TTACATGAAAGGATCTTCAATGCC
DhelOBP5-HF	TATTTTCAGGGATCCGAATTC CTCACTGATGAACAAAAGGAACGT
DhelOBP5-HR	GTGGTGGTGGTGGTGCTCGAG TTAAGAAGTAAGGCTTATGTGAGTAGGAG
DhelOBP6-HF	TATTTTCAGGGATCCGAATTC TTAACAGATGAACAAAAGGAGAAAATT
DhelOBP6-HR	GTGGTGGTGGTGGTGCTCGAG TTAAGCTAAACTGATGTGTGTTGGTG
DhelOBP14-HF	TATTTTCAGGGATCCGAATTC CTATCAGATGAAATGAAGGAAGTCATT
DhelOBP14-HR	GTGGTGGTGGTGGTGCTCGAG TTAGAATAGCATGTAGTTATCTGGGTTG
DhelOBP18-HF	TATTTTCAGGGATCCGAATTC GACGAAATGAAAGAAGTTCTTCAGAC
DhelOBP18-HR	GTGGTGGTGGTGGTGCTCGAG TTAGAATAACATGTAGTTATCAGGATTATCTG
DhelOBP20-HF	TATTTTCAGGGATCCGAATTC GCCGAATTAACCCCAAAGGA
DhelOBP20-HR	GTGGTGGTGGTGGTGCTCGAG TTAGACATCCTTGGGGTATTCCA
